# A novel mechanical lung model of pulmonary diseases to assist with teaching and training

**DOI:** 10.1186/1471-2466-6-21

**Published:** 2006-08-20

**Authors:** J Geoffrey Chase, Toshinori Yuta, Kerry J Mulligan, Geoffrey M Shaw, Beverley Horn

**Affiliations:** 1 Department of Mechanical Engineering, University of Canterbury, Christchurch, New Zealand; 2 Centre for Bioengineering, University of Canterbury, Christchurch, New Zealand; 3 Department of Intensive Care, Christchurch Hospital; Christchurch School of Medicine and Health Sciences, University of Otago, Christchurch, New Zealand

## Abstract

**Background:**

A design concept of low-cost, simple, fully mechanical model of a mechanically ventilated, passively breathing lung is developed. An example model is built to simulate a patient under mechanical ventilation with accurate volumes and compliances, while connected directly to a ventilator.

**Methods:**

The lung is modelled with multiple units, represented by rubber bellows, with adjustable weights placed on bellows to simulate compartments of different superimposed pressure and compliance, as well as different levels of lung disease, such as Acute Respiratory Distress Syndrome (ARDS). The model was directly connected to a ventilator and the resulting pressure volume curves recorded.

**Results:**

The model effectively captures the fundamental lung dynamics for a variety of conditions, and showed the effects of different ventilator settings. It was particularly effective at showing the impact of Positive End Expiratory Pressure (PEEP) therapy on lung recruitment to improve oxygenation, a particulary difficult dynamic to capture.

**Conclusion:**

Application of PEEP therapy is difficult to teach and demonstrate clearly. Therefore, the model provide opportunity to train, teach, and aid further understanding of lung mechanics and the treatment of lung diseases in critical care, such as ARDS and asthma. Finally, the model's pure mechanical nature and accurate lung volumes mean that all results are both clearly visible and thus intuitively simple to grasp.

## Background

Physiological modelling with mechanical and/or mathematical models has been a major focus for many bioengineering researchers. Such models are useful in understanding the physiological function or process, and can lead to development of new treatments or strategies. A model can also be used to simulate and predict a body's reaction to certain treatment, drugs, or dosages without actually testing it on a patient. Furthermore, these models can simulate weeks' worth of treatment in a compressed time, aiding the learning process and ultimately improving clinical decisions at the bedside.

The difficulty of representing actual physiological function and process is that the body can be highly non-linear. In many cases, the exact mechanics are not well known, and the same physiological response to an event can vary greatly depending on the individual, condition, timing, and other factors. Therefore, any model needs to capture enough characteristics for it to be clinically useful, while considering minimal number of parameters and simplified dynamics to be practical. Determining the content of this minimal set is the crux of the model design problem, and is particularly difficult for purely mechanical model.

This issue is not unique to mathematical or physical models alone. Many models use computer programming, sensors and actuators to ensure a physiologically accurate model response where mechanics may be lacking [1]. This approach is common in lung modelling for training system [2,3]. In particular, Verbraak et al [4] uses a computer software to define lung properties of the simulator. Similarly Mesic et al [5] uses feedback control system to operate mechanical components and achieve the desired results. Meka et al [6] have developed bellow-less lung simulator to simulate spontaneous breathing. However, it uses sensors and computers extensively in a similar fashion to achieve the desired effect. As a result, they are more complex and expensive, providing a primarily programmed response. More importantly, the model becomes more of a black box losing clarity in illustrating the inner working dynamics that represent the physiology.

A majority of patients admitted to an Intensive Care Unit (ICU) require mechanical ventilation (MV) [7]. However, ventilation settings are usually determined by trial and error using heuristic rules due to difficulty in understanding the interaction between ventilator and patient specific fundamental lung mechanics. Hence, ventilator treatment is largely influenced by the experience and intuition of the intensive care staff as seen by the wide variety of different published protocols [8-12].

Suboptimal ventilator strategies are not as crucial in patients with near-normal lung function who only require basic support while sedated. However, injured lungs, such as in Acute Respiratory Distress Syndrome (ARDS), are less forgiving. The lack of understanding is especially pronounced in such diseased lungs, where ventilator therapy is crucial to recovery and outcome, and is focused on safely maximizing recruitment of lung units to maximize oxygenation.

New studies suggest alveolar lung unit recruitment and derecruitment are the major cause of lung volume change in ventilated breathing, rather than balloon-like expansion of these alveolar lung units [13,14]. The pressure-volume relations of the lung is thus characterized by Threshold Opening Pressure (TOP) and Threshold Closing Pressure (TCP) for these units. TOP and TCP are the critical pressures at which lung units open and collapse, respectively, and whose values depend on superimposed pressure and condition. These mechanics have been use in a mathematical model and proven useful for evaluating the mechanical ventilator treatment [15,16].

Ventilator therapy is primarily based on optimizing TOP and TCP to improve recruitment and oxygenation. Therefore, a model, especially for training, must capture this behaviour accurately and in a clearly visible way. To date, no purely mechanical lung model, particularly with physiological volume and mechanics, can accomplish this goal.

In a diseased state, the compliance of the lung changes significantly. For example, in ARDS lung compliance decreases due to inflammation and swelling, or edema, of the tissues [17] and denaturalization of surfactant causing increased surface tension. Such impaired lungs require higher pressure to open. Increased pressure is required to displace fluid and overcome the additional weight of fluid, oedematous tissue, and surface tension. If the additional required pressure is too great, some lung units collapse on expiration and cannot be recruited during subsequent inflation, reducing the functional volume of the lung [18,19] and decreasing oxygenation of the blood. This impairment especially affects the bottom, more dependent region, of the lungs, where the weight of the lung above acts as additional weight, or superimposed pressure [20].

The overall effect of ARDS is a volumetrically smaller and stiffer lung. Poor ventilator strategy for such patient result in less recruitment of lung units, which leads to low perfusion and reduced gas exchange, or over distention of lung units. These in turn can lead to further injury of the lung. Thus, a choice on ventilator strategy has significant effect on outcome of the patient.

In clinical practice, Positive End-Expiratory Pressure (PEEP) is one of the key interventions in treating patients with lung disease. PEEP prevents alveolar collapse by compensating for the increased closing pressure in ARDS. Positive pressure keeps the lungs partially inflated at the end of expiration, thus maintaining a higher number of functional lung units [21,22].

In theory, higher PEEP recruits and maintains more functional lung units, leading to increased oxygenation of the blood. However, for a constant tidal volume (max-min volume), higher PEEP also increases Peak Inspiratory Pressure (PIP) and the overall driving pressure (PIP-PEEP). Increasing PIP too much, by simply increasing the PEEP, over-distends the lungs, causing further barotrauma. In contrast, too little PEEP and repeated collapse of ARDS affected units at the end of each breathing cycle will also lead to further damage.

Setting PEEP is thus a delicate clinical balance between maximizing recruitment and minimizing damage due to units repeatedly collapsing at too low a PEEP or barotrauma at too high a PEEP. Setting PEEP is a matter of experience and subject to considerable variability in treatment and thus outcome [23]. Lack of a complete understanding of lung mechanics can exacerbate this difficulty.

This paper presents a design of completely mechanical model of the human lung to identify and verify the fundamental mechanics of any mechanically ventilated lungs and the effect of PEEP on recruitment. The model can be connected directly to a standard ventilator, thus physiologically accurately representing a fully mechanical ventilated patient. As a result, it can be used for teaching and better understanding lung mechanics, as well as testing ventilator strategies and different (modelled) patient types, leading to improved treatment of ventilated patient. Its completely mechanical nature and simple mechanism clearly illustrate the behaviour of different lung volumes and their interaction with the ventilator settings.

## Methods

A model was built using mechanical components to represent the passively breathing, mechanically ventilated lung. The model captures the basic mechanics of the lung including realistic compliance, resistance, and overall volume. The compliance and the resistance can be fully and easily adjusted to simulate different lung disease states, such as ARDS, and obstructive lung diseases or conditions, such as asthma. Volume restricting mechanics, such as the effect of the chest wall and elastic limits of the connective tissues, were included to control the maximum volume, and are also adjustable.

The effect of superimposed pressure is simulated by using different weights positioned on top of each bellows. Heavier weights are used to simulate the dependent region, where the effect of the weight of the overlaying lung results in the greatest superimposed pressure. Non-dependent regions are simulated by using lighter weights because they are least affected by the weight of the overlaying lung [20]. A stiffer ARDS or disease affected lung can be modelled similarly by using added weight.

Particular focus was placed on accurately capturing the effects of:

1. Flow resistance.

2. Recruitment and derecruitment of lung volume.

3. Superimposed pressure due to lung region or disease state.

These dynamics represent the major, fundamental aspects involved in understanding and effectively managing ICU ventilator treatment for ARDS and other severe lung diseases.

### Model

**Figure 1:**
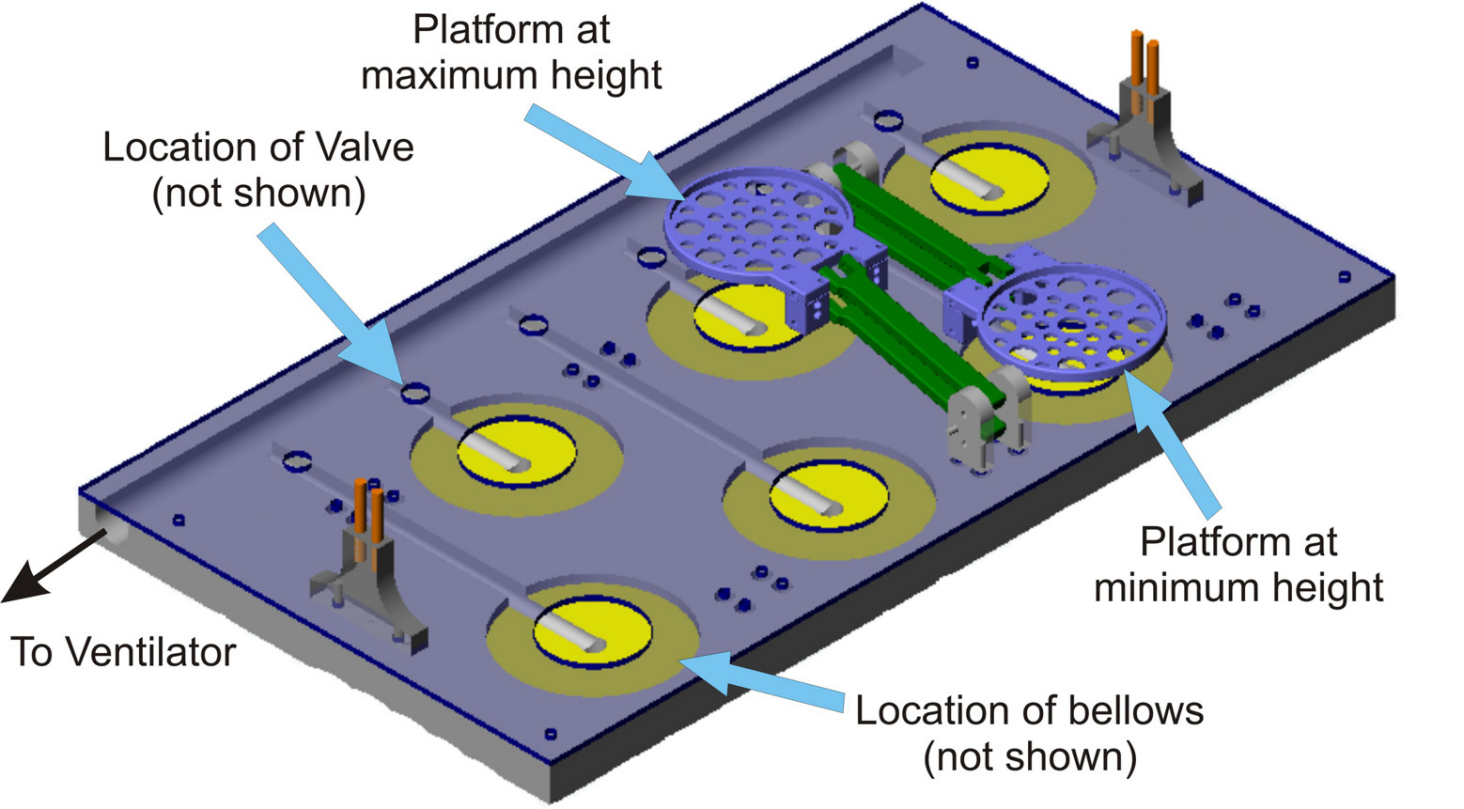
**A 3D drawing of the model**. The overall view of the model including relative locations of the bellows and the valves, as well as the position of weight platforms. Only 2 platform/supporting arm assemblies are shown for clarity.

The mechanical model of the lung was built using simple components without electronics or actuators. The goal was to simulate the physiological lung completely mechanically. In addition, a simple model can be easily and cheaply recreated. Each component was designed to represent a specific function within the fundamental mechanics of the lung. Figure 1 shows a 3D design drawing of the system designed.

The lung volume is represented by 6 latex rubber bellows. The number of bellows was chosen for size limit of the model and practicality of construction, while maximizing the accuracy and resolution of results. Fully inflated, each bellow contain approximately 200 ml of air before the rubber starts to stretch, resulting in a maximum lung volume of 1200 ml above functional residual capacity (FRC). FRC is the minimum lung volume under normal breathing. This model simulates the normal breathing lung. Thus, volume below FRC does not need to be simulated. The zero volume of the model thus equates to FRC of the simulated lung.

**Figure 2:**
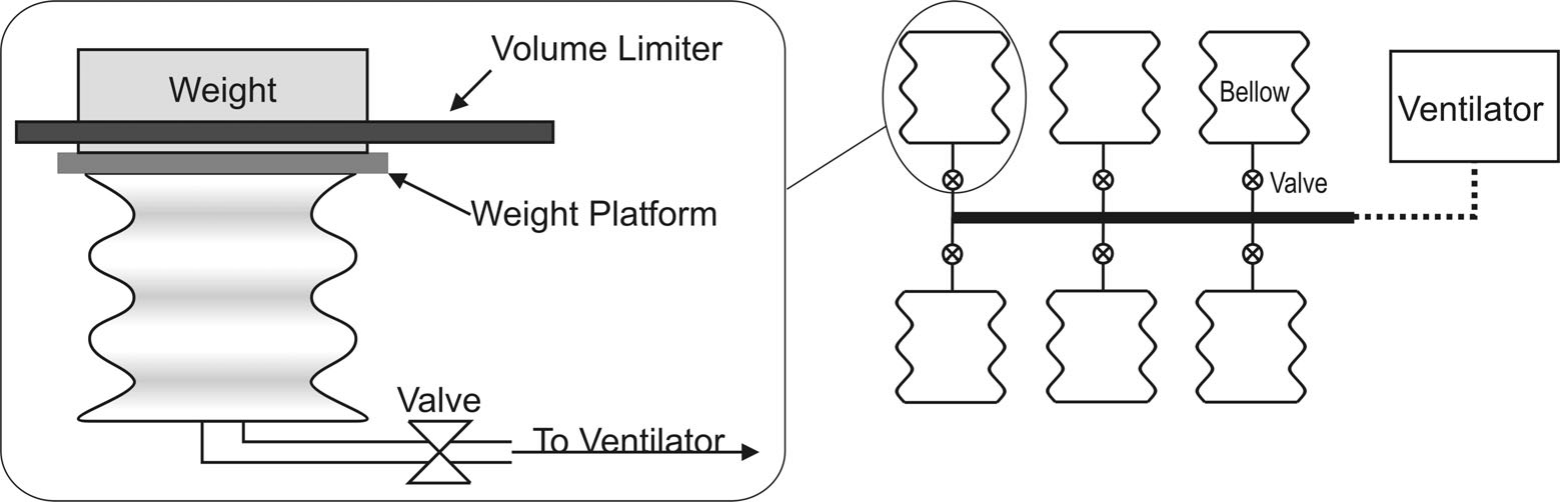
**Schematic drawing of model**. The lung is represented by 6 bellows connected to a ventilator. A platform is placed on top of rubber bellow to support weights, used to vary the driving pressure of the bellow. A volume limiter sets the maximum height of the platform, thus limits the height which the bellow is allowed to expand vertically. Each bellow is connected to the larger common tube through an adjustable valve.

Each bellow is connected by a 4 mm inner diameter air hose in the base to a larger 13 mm inner diameter common tube through a valve. The valve can be adjusted continuously to vary the orifice resistance to capture characteristics of obstructive pulmonary diseases, such as asthma. The larger tube is then connected to the ventilator. A schematic drawing of the model is shown in Figure 2.

A metal platform is placed on top of each bellow. The platform is supported by metal arms to support the weights used to simulate different superimposed pressure and lung disease states. The amount of weight can be varied to represent different levels of super-imposed pressure in different compartments of the lung, as well as compliance of each units. Additional weight can be used to simulate the local disease state or condition, where more weight would represent stiffer lung, or a lower compliance, requiring extra pressure for recruitment.

Each bellow represents a lung unit, which consists of all the distal branches of the bronchial tubes and alveoli for that lung volume. Thus, this model can simulate a lung with up to 6 compartments of different super-imposed or diseased state based on opening pressure. The design can be thus readily expanded to a greater number of smaller volume bellows to provide a greater number of compartments or disease states.

A valve is placed between each bellows and the larger common tube. In the fully open position, the opening of the valve has a similar diameter as the tube, thus the valve creates no significant additional resistance. The valve can be adjusted continuously. As the valve is closed, the effective airway diameter is reduced and the resistance to flow increases. At the fully closed position, the air flow is completely shut down. Physiologically, the valve represents the effective airway diameter between the trachea and bronchial tubes. The valves are also used to vary the resistance to the air flow to account for different sizes, obstruction or characteristics of airways.

An adjustable volume limiter is implemented by restricting the maximum height of the platform. The limiter simply prevents the bellow from further vertical expansion at a user-defined volume. Once the set limit is reached, the volume of the bellow can increase only by stretching the rubber outward, significantly increasing the resisting stiffness of the bellow. The volume limiter represents physical constraints, such as the chest wall or tension of the alveoli wall and connective tissues at maximum expansion.

Since each bellows unit can expand independently, when one unit reaches the limiter, air flow and motion will transfer to a path of lesser resistance in other units that may still be free to expand. Hence, the least weighted and lowest resistance units expand first, which matches physiological expectations of recruitment [20]. Similarly, stiffer units with larger weights and opening pressures are recruited last, also matching clinical observation.

**Figure 3:**
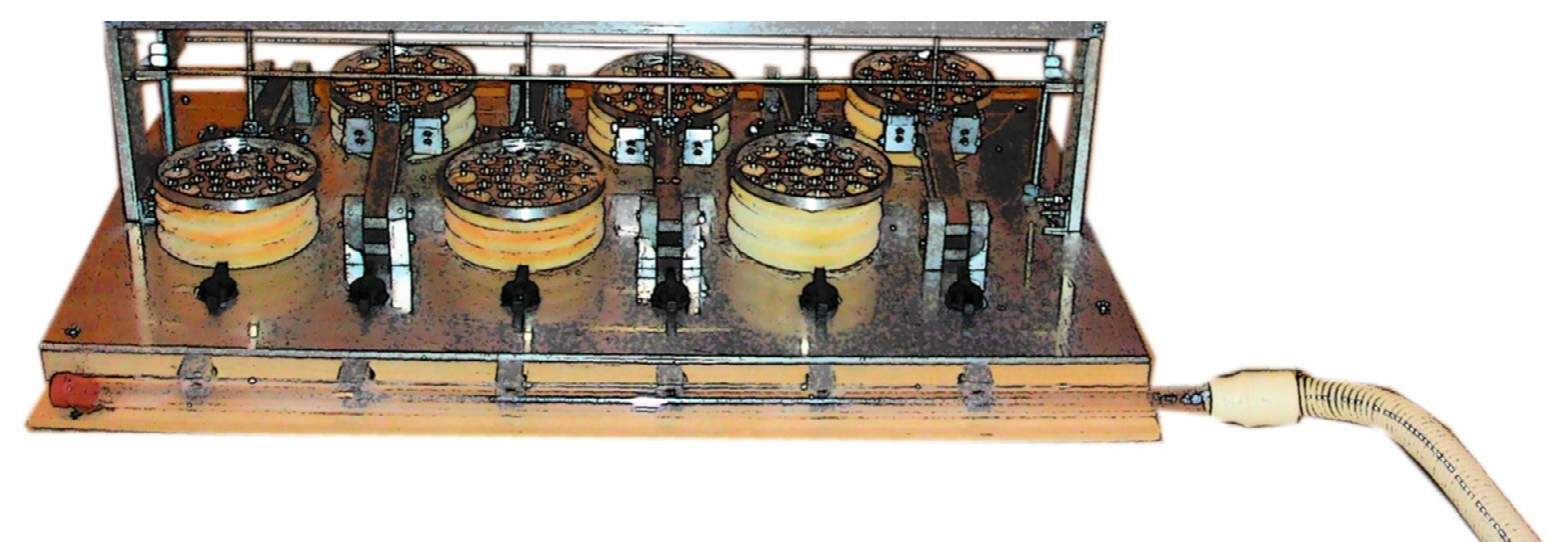
**Mechanical lung model including the connection to the ventilator**. Each bellows, or lung unit, is connected to a larger common tube, shown in front, through an adjustable valve, which can be seen in front of each bellow.

**Figure 4:**
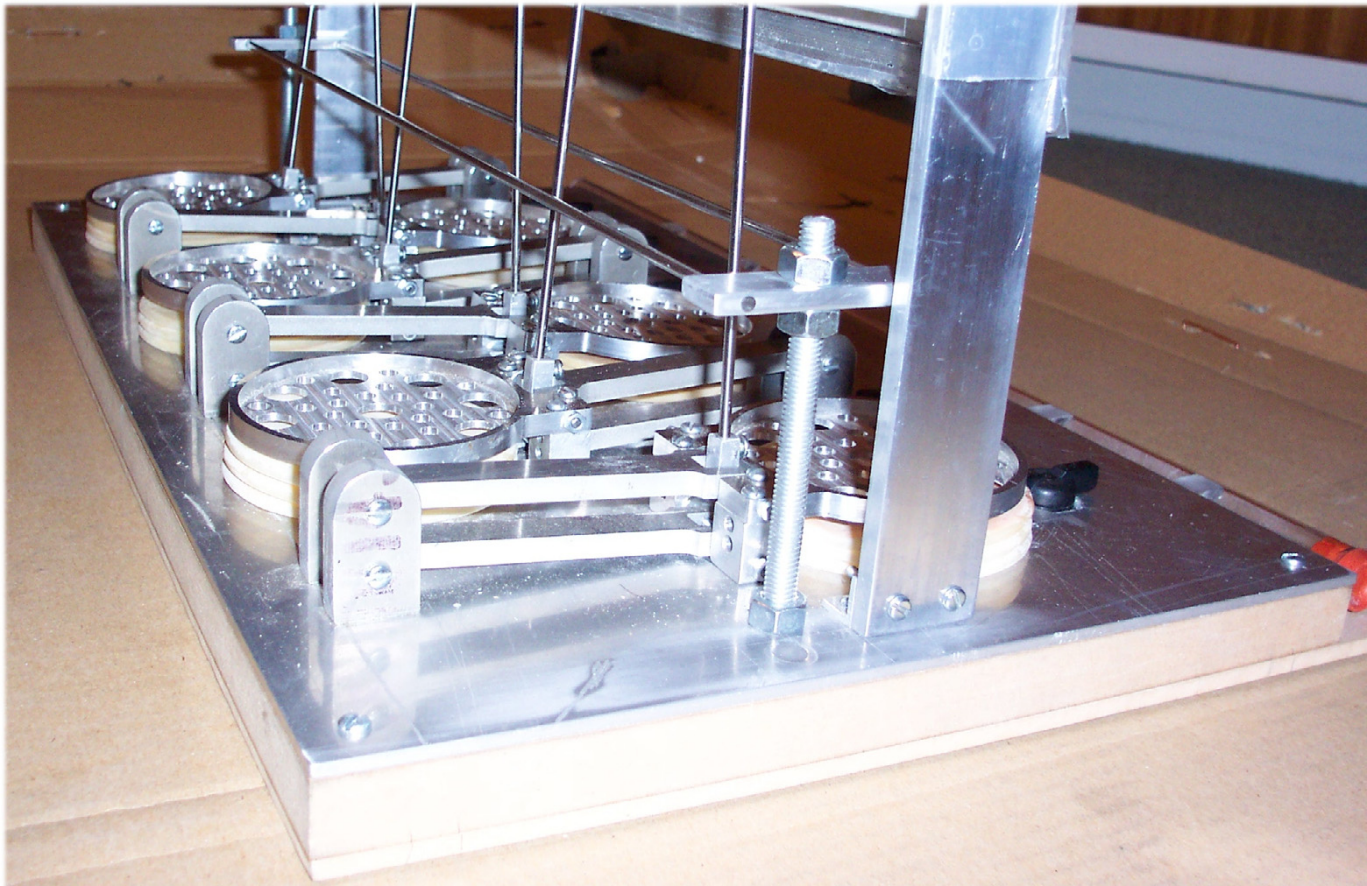
**Model components**. The figure shows the close up of mechanical components. A metal platform is placed on top of each bellows, support by arms. A volume limiter is implemented as two metal rod placed horizontally above the platforms.

Figure 3 shows the overall view of the mechanical model including the connection to the ventilator. The model, as shown, has no extra weight and is partially expanded. Figure 4 shows another clear prospective view of the model.

### Testing method

A Puritan Bennett 840 ventilator was used to ventilate the model. The model was adjusted to simulate different patient conditions and disease states. The resulting pressure-volume curves were obtained directly from the ventilator and recorded to verify that the fundamental model mechanics were accurate.

The model inflates in two phases during inspiration. In the first phase, the air fills the bellow while it expands vertically without stretching the rubber. This phase represents the recruitment of previously collapsed, or partially open, lung units under the pressure modelled by the weights on top of the bellow. The compliance of this phase is regulated by the TOP and TCP. In the model presented, it is controlled by the weights on the unit, preventing expansion until greater pressure is available. However, the result is the same as units open and close at specific threshold pressures.

The second phase starts when a bellow reaches maximum height and the volume limit. The bellow starts to stretch in this phase and represents inflation of already recruited lung units. In the model representing a healthy lung, this second phase does not begin until all the other units have reached their maximum limit. Thus, bellows stretching represents over distension of the lung, or clinically, barotrauma.

In the diseased or severe ARDS lung, this distension can begin before all units are recruited [24]. For example, consider a lung unit with TOP of 5 cmH_2_O and severely ARDS affected unit with a TOP of 15 cmH_2_O. If the driving pressure is set from 5 to 15 cmH_2_O, by the time the Severely affected unit can be recruited, the other unit with lower TOP is over distended. This behaviour represents a common tradeoff in optimizing ventilator settings, particularly where direct measurement of recruitment or lung unit status are unavailable. Thus, clinicians effectively operate blind in this regard. Overall, the model easily captured this tradeoff, and shows how it occurs much more clearly and readily then when dealing with a clinical case where barotrauma is hard to measure or identify from the observations and measurements clinically available.

A weight placed on the bellow platform was used to control the opening pressure for the bellows, simulating the compliance of that part of the lung. The weights can be incrementally increased across the 6 bellows to represent the increasing level of superimposed pressure from the top to the bottom of the lung. The less-dependent region of the lung is affected least by the weight of the rest of lung, thus the superimposed pressure is low and no additional weight is put on the bellow. The more dependent region is affected most by the weight of the rest of lung, and the superimposed pressure at this level can be as high as 20 cmH_2_O.

In severe lung disease, such as ARDS, the compliance of the lung decreases significantly due to the higher pressures required to recruit ARDS affected lung units. This increase in required pressure can easily be simulated by increasing the amount of weight for any given units. The dependent region of the lung may collapse completely in ARDS, removing these units from contributing to any volume change, particularly if they are affected enough not to be recruited at high ventilator pressure. This situation can be simulated by drastically increasing the weight for the bellow. Cups of water can be used as continuously adjustable weight, which visually represent the additional pressure. This approach is intuitively instructive as the water can be measured in height, cmH_2_O, which is a direct pressure measurement used clinically.

ARDS affected lung is stiffer than healthy lung. This decreased compliance is represented by adding extra weights for the affected units. The additional weights can be distributed to all units to simulate the homogenous lung, or placed only on some of the units to simulate heterogenous lung, both of which are observed in ARDS affected lung [19]. Hence, for example, the additional weights can be placed on 3 bellows to represent ARDS affected units and the other 3 without additional weights to represent healthy units. This case simulates a heterogenous lung with 50% ARDS affected lung units. The location of the ARDS affected units in the lung can be varied by placing the weights on the bellows representing different levels of superimposed pressure.

The valves can be used to simulate resistive diseases and conditions of the lung, such as asthma. Different levels of severity for each compartment can be simulated by individually adjusting the valves for each bellow. However, the variable valves do not simulate dynamic airflow obstruction, such as increased resistance on expiration.

### Validation experiments

The model was connected directly to a ventilator (840 Ventilator System, Puritan Bennett, Pleasanton, CA, USA). The weight for each lung unit was incrementally increased to simulate superimposed pressure of 2 to 15 cmH_2_O. The volume limit of each bellow was set to 200 ml above functional residual capacity. This allowed a maximum tidal volume of 1200 ml. The ventilator was then, turned on to let model "breathe". PV curves and movement of the bellows were observed and recorded for different PEEP levels, and compared to clinical expectations.

## Results

**Figure 5:**
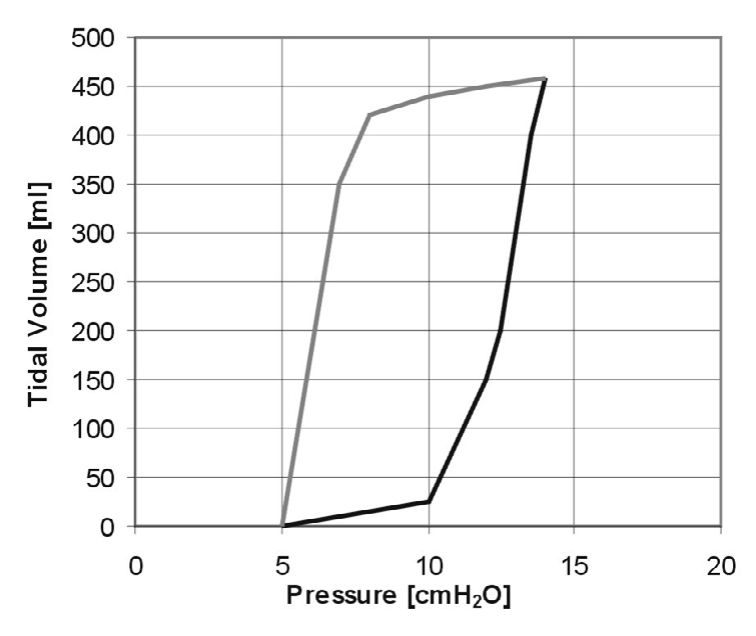
**Normal lung**. PV loops produced by model with PEEP at 5 cmH_2_O and Tidal volume of 450 ml. The plot shows a typical shape of PV curve produced by the model. Both inflation and the deflation limb follow the general trend of sigmoid curve.

**Figure 6:**
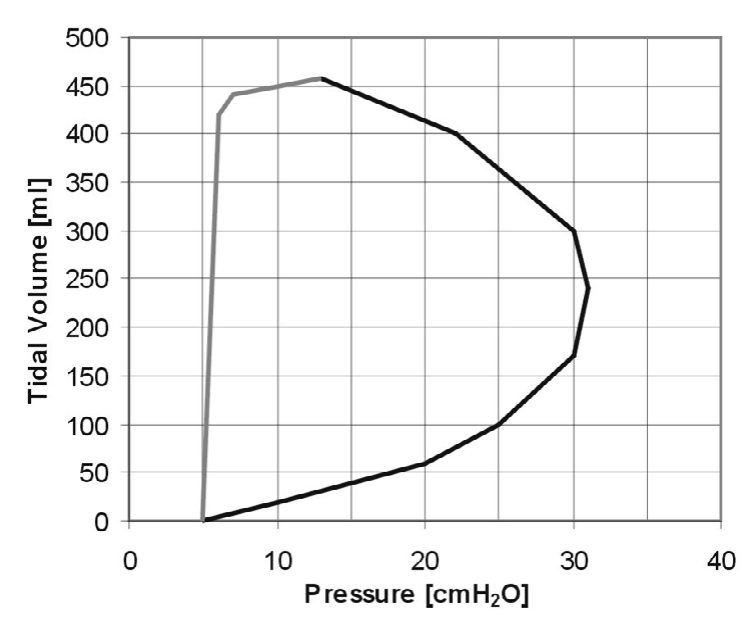
**Obstructive disease. i.e. asthma**. PV loops produced by model with PEEP at 5 cmH_2_O and Tidal volume of 450 ml. The plot shows PV curve produced by the model with increased resistance, simulating a flow restricting disease, such as asthma.

Overall, the model produces physiological PV curves with approximately sigmoid inspiratory and expiratory limb at any value of PEEP, as shown for one PEEP value in Figure 5. It shows low compliance at low and high pressures, and relatively high compliance in the middle, more linear portion. Figure 6 shows the PV loop for similar settings with increased airway resistance, simulating the same lung with a obstructive disease, such as asthma. The inspiration limb in Figure 6 shows that the pressure increases initially without much increase in volume. During the inflation phase, the gas is pushed into the larger tube, however, due to the small opening of the valve, the flow is restricted and the gas cannot enter the bellow readily. As a result, the pressure builds up within the tube, producing the initial, less compliant, segment of the PV curve. As the pressure gradient increases across the valve, the steady flow is established and the volume slowly "catches up" as the bellows are inflated, and the pressure across the valve equalizes. This behavior matches clinical expectation for obstructive disease such as severe asthma.

**Figure 7:**
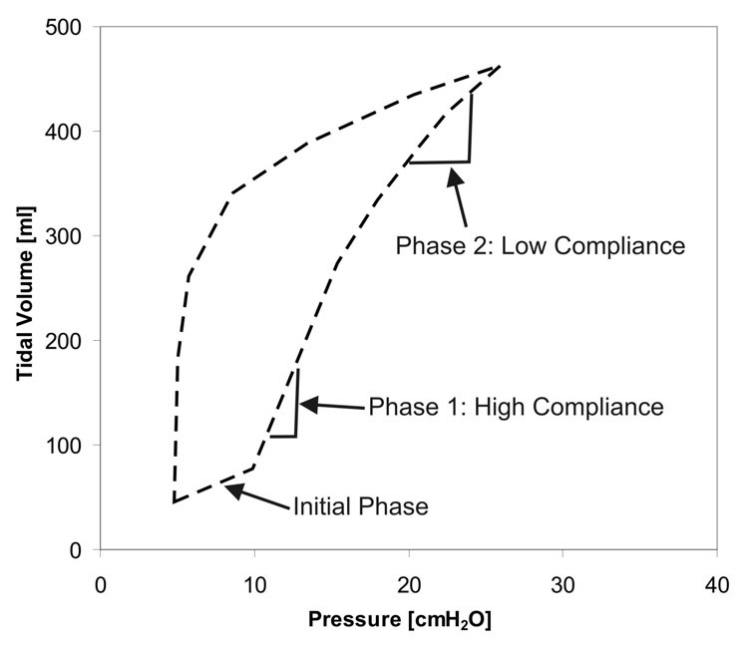
**Compliance for different phases**. Most of volume change occur in first phase, where bellows are expanding vertically without stretching rubber, resulting in higher compliance. Second phase occurs when the bellow platform reaches maximum height and start to stretch the rubber, resulting in lower compliance. The initial phase is where pressure increases in tube before the air starts to flow into the bellows.

**Figure 8:**
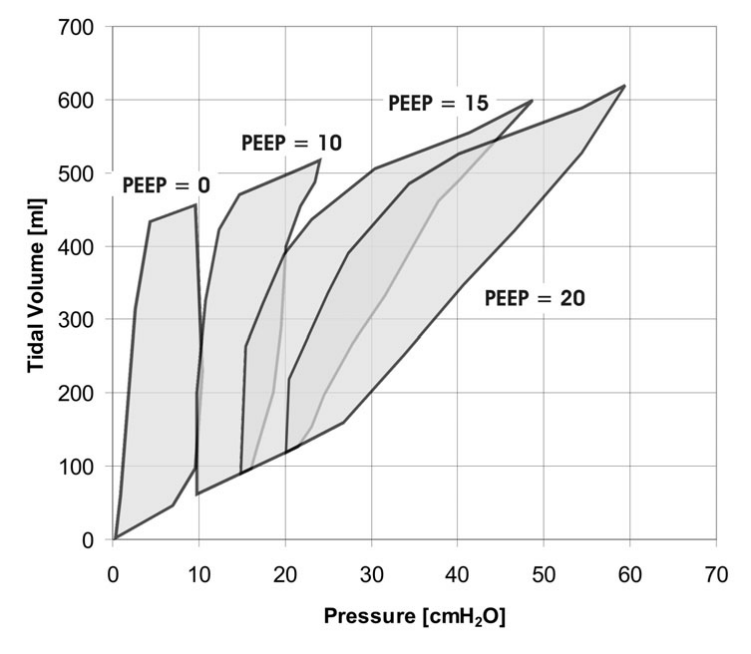
**PV curves for different PEEP**. The above plot shows the differences in the shapes of PV loops with different PEEP level and same tidal volume. The plot shows the tidal volume and estimated FRC. As can be seen, the compliance gradually decreases as PEEP is increased.

Most of the volume change occurs in the first phase, as shown in Figure 7, while the bellows are expanding vertically. The second phase results in a relatively smaller volume change for a given pressure change as the bellows are stretched. The first phase resulted in higher compliance, and the second phase resulted in relatively low compliance as, expected during over-distension. This behaviour is more evident as PEEP increases causing greater amount of over distention units and thus modelled barotrauma, as shown in Figure 8. More specifically, increasing PEEP leads to greater second phase response and lower average compliance over the inspiration limb.

With a Zero End-Expiratory Pressure (ZEEP) setting or PEEP = 0 cmH_2_O, all the units are deflated completely at the end of deflation, and only the less-dependent units are inflated using same driving pressure. At relatively low PEEP levels, the least dependent units, with least superimposed pressure are partially inflated at the end of deflation resulting in non-zero volume at the end of expiration. In addition, more dependent units, with higher superimposed pressure, start to inflate during breathing. As PEEP is increased, more units remain partially inflated at the end of expiration and more dependent units are inflated during the breathing cycle. This result represents the expected increased recruitment of lung units and thus lung volume as PEEP increases. At high PEEP, the less-dependent units were fully inflated throughout the breathing cycle and more dependent units were inflated.

Figure 8 shows PV loops produced by the model with the tidal volume set to 450 ml and the PEEP incrementally increased. The plot shows PV loops with estimated FRC. As can be seen in Figure 8, the compliance decreases as PEEP increases. At high PEEP, the units with low superimposed pressure are fully inflated throughout the breathing cycle, thus most of the 450 ml volume change occurs in units with heavier weights, the previously collapsed stiffer lung units. The lower compliance is also caused by the high pressure stretching the rubber of already inflated bellows, representing over distension of lung units.

## Discussion

The model effectively captures the fundamental mechanics of the passively breathing, mechanically ventilated lung, particularly the effect of PEEP on lung unit recruitment. The resulting PV curves closely model clinically reported curves, with 3 regions of different compliance: low compliance at low and high pressure with relatively high compliance in between, as seen in Figure 7 and 8. The region of low compliance at low pressure represents where the bellows have not started to expand, while the pressure in the tubes increase without a significant increase in volume. Once the pressure in the tubes reaches the critical level to overcome the weight on a lung unit, the bellow starts to expand and results in higher compliance. However, at higher pressure, the bellow start to stretch due to volume limiter or the additional weights, resulting in lower compliance. In physiological terms, this critical level of the pressure to expand the bellows represents the TOP of the lung units. The expansion of the bellows in first phase represents the recruitment of lung units and the stretching of bellows in second phase represent over distention of lung units.

The Recruitment, or the expansion of the bellows, occurs from the least dependent, least added weight, units to the most dependent, most added weight, units, as expected. This heterogeneous recruitment throughout the respiratory curve at different TOP values has been shown in several different studies using CT scans [20,25,26]. Thus, the model behaviour matches the clinically observed physiological response.

Clinically, PEEP is used to prevent lung units from collapsing on expiration, thus maintaining a higher level of functional lung units during the breathing cycle. This effect is clearly shown using this model with added weight to simulate diseased units and therefore matching clinical expectation [21]. More specifically, when PEEP was applied, the bellows of less dependent units did not deflate completely at the end of expiration, and more dependent, or disease affected, units are inflated only at end of inspiration. The overall effect is an increase in the total amount of recruited lung units during each breathing cycle at higher PEEP.

In theory, the higher the PEEP setting, the higher the level of recruitment and thus, the greater the number of functional lung units. However, in clinically determining the level of PEEP, the need to avoid over distension of already recruited lung units must also be considered. In healthy lungs, over distension is prevented by the presence of surfactant, which alters the surface tension according to the surface area to minimize the distension of a given lung unit and ensures even distribution of gas within a lung. In the diseased lung, such as with ARDS, the effect of surfactant is reduced and lung units are vulnerable to over stretching due to heterogeneous inflation. Thus, in a severely ARDS affected lung, PEEP high enough to recruit diseased units may have already over distended and damaged already recruited healthy units. Over distension is not completely caused by high PEEP, but by the resulting higher PIP for a breathing cycle with higher PEEP. Higher tidal volumes thus also can lead to over distension, and are typically minimized where possible.

This mechanism of over distension is also captured using this mechanical model. At lower PEEP, the bellows expand by vertical movement. As PEEP increases, the bellows representing less dependent units remain inflated at the end of expiration. When PEEP is increased further, those bellows expand by stretching the rubber, representing over distension of lung units. The resulting PV curve in Figure 8 shows the lower slope of the high PEEP settings compared to the low PEEP settings, illustrating over distension.

With certain weight combinations, the model can simulate over distended units in less dependent regions and completely collapsed units in more dependent regions occurring at the same time. Clinically, this situation occurs when high PEEP over distends healthy lung units while severely ARDS affected units remain derecruited. It is this trade-off between recruitment and over distension of lung units at different superimposed pressures and condition of disease that make the choice of the level of PEEP complex [24]. However, it is correctly and visibly obviously captured by this model. Thus, the model is physiologically representative of a wide range of the fundamental mechanics of mechanically ventilated lungs.

The example model presented utilizes 6 bellows to represent an entire lung, with each bellows representing a slice of the lung. Thus, the model is limited to 6 different compartments of superimposed pressure, and limited resolution for differentiating the healthy and disease affected lung units. Obviously, more bellows with smaller volume could be used for greater resolution, while maintaining the physiologically accurate mechanical behavior and lung volume.

Other lung simulators using approximately similar basic designs have been developed [4-6]. However, the majority of them rely on mechatronics and/or computers and sensors to control their parameters and dynamic response, and thus ensure a realistic result. While the software controlled mechatronics allows precise control of parameters and thus a simulation of a specific lung condition, it requires access to a computer, designated software and a power source. It also complicates the design, construction, and operation of the entire system, while also removing the visual, physical interpretation afforded by the design presented.

In contrast, simple purely mechanical design allows great portability, while effectively demonstrating the physiological mechanics of lung in a clear visual manner. However, it must be noted that this approach can be limited for more detailed research investigation. Hence, selection of such systems would depend on the desired usage, where the system presented offers more insight into the effect of disease state and compliance at low cost, and the more complex systems offer greater range of capability with less physical interpretations at potentially higher cost.

Based on this proven concept using expandable rubber bellows and weights, the model can easily be reproduced using readily available and manufacturable components. Its response is purely mechanical. Thus, the effects are visible and easily understood in during simulation as they are the result of these simple mechanics. Currently no available model has this capability and its low-cost design makes it readily reproduced.

## Conclusion

A purely mechanical, simple and low-cost model of the lung is developed based on fundamental mechanics and physiology. Simple rubber bellows are used to represent lung units with adjustable weights employed to control the compliance of each bellows and thus simulate superimposed pressure and disease state. The goal was to create a fully mechanical, physiologically representative system suitable for teaching, training, and ventilator equipment testing. This goal was achieved by directly connecting the model to a commercial ventilator without using software or other mechatronics to "simulate" the correct results in a clearly visible easily understood fashion.

The mechanically ventilated model inflates in two stages: vertical expansion of the bellows and stretching of rubber once a physiological volume limit is reached. The first expansion produces higher compliance than the second, as expected, and matches the physiological knowledge. Experiments were conducted using variety of combinations of weights to represent different condition and state of the lung. The model was directly connected to the ventilator, as would be a real patient, and the pressure and volume during breathing cycles recorded.

The model accurately illustrates the fundamental lung mechanics, especially the effect of PEEP on lung unit recruitment. It is also able to simulate the trade-off between level of recruitment and over distension of lung units. Even though the model is based on simple mechanics, it is able to simulate the ventilated lung response in variety of conditions, and shows effects of different ventilator settings. Thus, it can readily be used to aid further understanding of lung mechanics, and treatment of lung disease. Its design is also easily generated to include more lung units and bellows for greater resolution and more in-depth study.

## Competing interests

The author(s) declare that they have no competing interests.

## Authors' contributions

JC and GS conceived the study and coordinated the project. TY participated in construction of the model and drafted the manuscript. KM participated in designing and construction of the model. BH participated in drafting of manuscript.

## Pre-publication history

The pre-publication history for this paper can be accessed here:
